# Isothiocyanate NB7M causes selective cytotoxicity, pro-apoptotic signalling and cell-cycle regression in ovarian cancer cells

**DOI:** 10.1038/sj.bjc.6604778

**Published:** 2008-11-11

**Authors:** R K Singh, T S Lange, K K Kim, A P Singh, N Vorsa, L Brard

**Affiliations:** 1 Molecular Therapeutics Laboratory, Program in Women's Oncology, Department of Obstetrics and Gynecology, Women and Infants' Hospital, Brown University, Providence, RI 02905, USA; 2 Division of Biology and Medicine, Brown University, Providence, RI 02912, USA; 3 Department of Plant Biology and Pathology, Rutgers University, NJ 08901, USA

**Keywords:** isothiocyanates, NB7M, ovarian cancer, MAPK, apoptosis, cell cycle

## Abstract

The present report identifies indole-3-ethyl isothiocyanate NB7M as a potent cytotoxic agent with selective activity against cell lines derived from various tumour types. Ovarian cancer cell lines showed sensitivity to NB7M (60–70% cytotoxicity at 2.5 *μ*M), in contrast to control cells (TCL-1 and HTR-8; IC_50_ ∼15 *μ*M). In a screen performed by the National Cancer Institute (NCI) (NCI_60_ cancer cell-line assay) NB7M (NSC746077) reduced growth up to 100% with an IC_50_ between 0.1 and 10 *μ*M depending on the cell line studied. Using SKOV-3 ovarian cancer cells as a model, mechanisms of cytotoxicity were analysed. NB7M caused hallmarks of apoptosis such as PARP-1 deactivation, chromatin condensation, DNA nicks, activation of caspases-9, -8, -3, loss of mitochondrial transmembrane depolarisation potential and upregulation of pro-apoptotic mitogen activated protein kinases (p38, SAP/JNK). NB7M downregulated phosphorylation of prosurvival kinases (PI-3K, AKT, IKK*α*), transcription factor NF-*κ*B, and expression of DNA-Pk and AXL receptor tyrosine kinase. Subcytotoxic doses of NB7M inhibited DNA synthesis, caused G1-phase cell-cycle arrest and upregulated p27 expression. The present report suggests that NB7M is a selective cytotoxic agent *in vitro* for cell lines derived from ovarian and certain other tumours. In addition, NB7M acts as a growth/cell-cycle-suppressing agent and may be developed as a potential therapeutic drug to treat ovarian cancer.

In 2007 in the United States 1 444 920 new cancer cases were diagnosed and 553 888 patients died of cancer. Ovarian cancer is the leading cause of death from gynaecological malignancies and ranks second among newly diagnosed gynaecological cancers in the United States (Heintz *et al*, 2003; American Cancer Society, 2007). Although most patients (70–80%) initially respond to cytoreductive surgery and adjuvant paclitaxel and platinum-based chemotherapy the majority will experience disease recurrence (McGuire *et al*, 1996; Piccart *et al*, 2000). Re-treatment with a platinum-based drug is possible for some women the response rate to current second-line or third-line (after interim non-platinum therapy) chemotherapy is below 33% due to the rise of resistance to such drugs (McGuire and Ozols, 1998; Leitao *et al*, 2003; Lamberth *et al*, 2004; Ott and Gust, 2007). Therefore, the development of novel chemotherapeutics with increased activity and alternative modes of action to treat such tumours, instead of or in addition to or after platinum therapy, is desired.

Naturally occurring isothiocyanates (ITC) such as BITC, PEITC and sulforaphane have been shown to inhibit chemically induced tumorigenesis in animal models in the lung, stomach, colon, liver, oesophagus, bladder and mammary glands (Conaway *et al*, 2002). Mechanisms of ITC activity in cancer cells, such as induction of G2/M cell-cycle arrest, and apoptosis (Singh *et al*, 2004), suppression of angiogenesis with the disruption of microtubulin polymerisation and mitotic progression of endothelial cells (Jackson *et al*, 2007; Xiao and Singh, 2007), release of reactive oxygen species and disruption of mitochondrial membrane depolarisation have been described (Xiao *et al*, 2006). Isothiocyanates were shown to be substrates for human glutathione transferases (Kolm *et al*, 1995). In addition to various naturally occurring ITCs, synthetic ITCs such as E-4IB have been discovered, which sensitised ovarian cancer cells to cisplatin-induced apoptosis by affecting signalling pathways (Bodo *et al*, 2006).

In an initial attempt to design a more potent ITC class of antitumour agents, we recently screened novel indolyl ethyl ITCs for enhanced anticancer cell activity (Singh *et al*, 2007). 7Me-IEITC (methyl substitution at C7 of the indole moiety) is a key representative of this new generation of ITC with increased potency as compared to various naturally occurring ITCs such as BITC (Kalkunte *et al*, 2006), PEITC (Satyan *et al*, 2006) and sulforaphane (Singh *et al*, 2004). The mechanisms linked to this selective cytotoxicity include induction of apoptosis, alteration of mitogen activated protein kinase (MAPK) signalling and cell-cycle inhibitory effects by 7Me-IEITC in both high-risk neuroblastoma (Singh *et al*, 2007) and platinum-resistant ovarian cancer cells (Singh *et al*, 2008).

The primary objective of this study was to further optimise the structural attributes of 7Me-IEITC (Figure 1A). The rationale of adding a tert-butyl carbamate group (converting the compound into NB7M; Figure 1A) was to protect the amino group in the hope of increasing the bioavailability as the lipophilic protection of a nitrogen atom in various anticancer drugs enhances tissue permeability (Serova *et al*, 2007). In a recent study, we reported an increased cytotoxicity and rapid induction of apoptosis by NB7M in nervous system cancer cells *in vitro* (Brard *et al*, 2008). In the present study, we (1) compared the cytotoxic effects of NB7M on ovarian and other tumour-derived cell lines, (2) identified the mechanisms of programmed cell death of SKOV-3 cells induced by NB7M (3) analysed the expression of key MAPKs and other prosurvival markers and (4) reported inhibitory effects of subcytotoxic doses of NB7M on cell-cycle progression of SKOV-3 cells substantiated by studies on the expression of check-point regulators of the cell cycle.

## Materials and methods

### Compound

Details to the synthesis and structural characterisation of NB7M and the standardised NCI_60_ cancer cell-line *in vitro* assay are described in the Supplementary Information section. The purity and stability of NB7M was determined by high performance liquid chromatography. Purity of NB7M was 98%; NB7M showed stability in solution for 72 h at RT and of more than 12 weeks (period of investigation) at −20°C.

### Cell culture

Cell lines SKOV-3, OVCAR-3 (human ovarian epithelial adenocarcinoma), PC-3 (human prostate adenocarcinoma), BxPC-3 (human pancreatic adenocarcinoma) and A-431 (human epidermoid skin carcinoma) were obtained from American Type Culture Collection (Manassas, VA, USA). TCL-1 (human immortalised retroviral large T-antigen transfected trophoblasts) and HTR-8/SVneo (first-trimester cytotrophoblasts with extended lifespan) were kindly provided by Dr Surendra Sharma, Providence, RI, USA. All cells were seeded at 5 × 10^5^ per flask and cultured to ∼80% confluency in T75 cell culture flasks (Corning, New York, NY, USA) in DMEM or RPMI (Gibco, Rockville, MD, USA) supplemented with 10% fetal bovine serum (Atlanta Biologicals, Lawrenceville, GA, USA), 100 U ml^−1^ penicillin and 100 *μ*g ml^−1^ streptomycin at 37°C, 5% CO_2_, in a humidified incubator.

### Cell viability assay

Viability of cells was determined by the 96®Aqueous-One-Solution Assay (Promega, Madison, WI, USA) (Malich *et al*, 1997). For assays with inhibitors (p38/SB203580, caspase-3/Z-DEVD-FMK, Calbiochem, La Jolla, CA, USA) cells were pre-incubated with 40 *μ*M inhibitor for 2 h before drug addition. The assay was carried out as described previously (Lange *et al*, 2007); data are expressed as the mean of the triplicate determinations (X±s.d.) of a representative experiment in % of absorbance of samples with untreated cells (100%).

### Mitochondrial transmembrane depolarisation potential

Cells (1 × 10^6^) were seeded in a 100 mm^2^ Petri dish in DMEM complete media and incubated for 24 h. Media was removed and replenished with fresh media containing 2 *μ*M NB7M for 12 or 24 h. The test was carried out as described previously (Singh *et al*, 2008). Ten thousand cells were analysed for each sample.

### Morphological studies

Cells were seeded into a Lab-Tek Chamber Slide System. (Nalge Nunc., Int., Naperville, IL, USA) at a concentration of 1 × 10^4^ per chamber in complete medium, incubated overnight, and treated for 24 h with 2 *μ*M NB7M (∼IC_50_) at 37°C, 5% CO_2_. The test was carried out as described previously (Lange *et al*, 2008). Representative images were taken with an inverted microscope (Nikon Eclipse TE2000-E fitted with a cooled CCD camera) and × 20 objective.

### Cell proliferation assay

Cell proliferation was determined by a BrdU assay (Roche Applied Science, Indianapolis, IN, USA) measuring the incorporation of the pyrimidine analogue, 5-bromo-2′-deoxyuridine (BrdU) during DNA synthesis. Briefly, cells (5 × 10^3^) were plated into 96-well flat bottom plates (Corning Incorporated, Corning, NY, USA) and allowed to attach overnight before treatment with NB7M (results section) for 42 h in complete medium. The assay was carried out as described previously (Lange *et al*, 2008). Experiments were performed in triplicates; data are expressed as mean of triplicate determinations (X±s.d.) of a representative experiment in % of absorbance of samples with untreated cells (100%).

### Cell-cycle analysis (by FACS)

Cell-cycle analysis and quantification of apoptosis were carried out by flow cytometry. Cells were seeded into 100 mm^2^ tissue culture dishes (7.5 × 10^5^ cells per dish, Corning Incorporated), allowed to attach overnight and treated in complete medium under cell culture conditions with 2 *μ*M NB7M for 24 or 48 h. The test was carried out as described previously (Lange *et al*, 2008). Ten thousand events were analysed for each sample. Appropriate gating was used to select the single-cell population. The same gate was used on all samples, ensuring that the measurements were made on a standardised cell population.

### TUNEL assay

DNA fragmentation was detected by terminal deoxynucleotidyl transferase-mediated dUTP nick-end labelling (TUNEL), using the DeadEnd™ Fluorometric TUNEL System assay (Promega, Madison, WI, USA). Briefly, cells (5 × 10^3^) were plated into 96-well flat bottom plates (Corning Incorporated) and allowed to attach overnight before treatment with 2 *μ*M NB7M or 25 *μ*M actinomycin D for 24 h in fresh complete medium. The assay was carried out as described previously (Lange *et al*, 2008). All experiments were performed in triplicate.

### Western blot analysis

Cells were seeded in 100 mm^2^ tissue culture dishes (5 × 10^5^ cells per dish) and cultured to ∼80% confluency. Cells were treated with or without 2 *μ*M NB7M for various lengths of time (result section). Polyacrylamide gel electrophoresis and immunoblotting were carried out as described previously (Lange *et al*, 2008). Primary antibodies (Cell Signaling Technology, Beverly, MA, USA) were diluted 1 : 1000 in PBST/5% BSA. Bands were visualised using horseradish peroxidase-conjugated secondary antibodies (Amersham-Pharmacia Biotech, Piscataway, NJ, USA), followed by enhanced chemiluminescence (Upstate, Waltham, MA, USA) and documented autoradiography (F-Bx810 Film, Phenix, Hayward, CA, USA).

## Results

### NB7M shows differential effects on the viability of various human cancer cell lines

**Figure 1:**
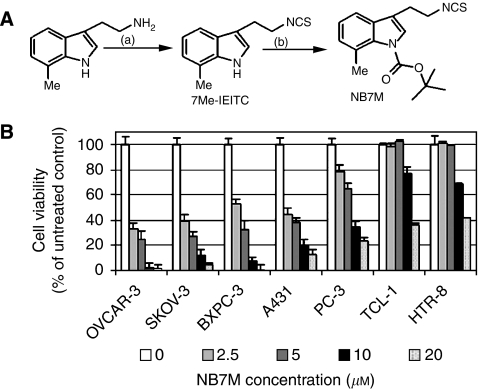
Design concept and cytotoxicity of indolyl ethyl isothiocyanate NB7M. (**A**) Synthesis and structure of 7Me-IEITC derivative NB7M. (see Materials and Methods; Supplementary Information). (**B**) Comparative analysis of the cytotoxic effect of NB7M in various human cancer and control cell lines. SKOV-3, OVCAR-3 (ovarian epithelial adenocarcinomas), PC-3 (prostate adenocarcinoma), BxPC-3 (pancreatic adenocarcinoma), A-431 (epidermoid carcinoma), HeLa (endometrial), TCL-1 (trophoblasts) and HTR-8 (first-trimester cytotrophoblasts) human cell lines were treated with various concentrations (0–20 *μ*M) of NB7M for 48 h. The MTS viability assay was carried out as described (Materials and Methods). Experiments were performed in triplicates; data are expressed as the mean of the triplicate determinations (X±s.d.) of a representative experiment in % cell viability of samples with untreated cells (100%).

In an initial approach to analyse the effects of NB7M (Figure 1A) on ovarian cancer cells, we performed a cytotoxicity assay (Figure 1B) using SKOV-3 and OVCAR-3 (human platinum-resistant ovarian epithelial adenocarcinoma) cell lines in comparison to adenocarcinoma cell lines from different tissues (e.g., BxPC-3, pancreatic; PC-3, prostate). Furthermore, we tested the effect of NB7M on A-431 cancer cells (human epidermoid) and immortalised cell lines with primary features (TCL-1, trophoblasts; HTR-8, first-trimester cytotrophoblasts).

**Figure 2:**
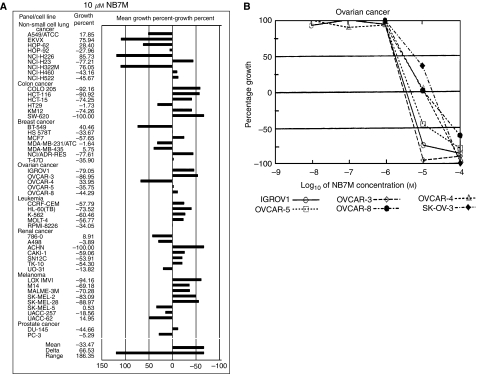
NB7M effect on cell growth in NCI_60_ cancer cell line screen. (**A**) NCI_60_ cell line *in vitro* screening at 10 *μ*M NB7M. Cells (see Supplementary Information) were treated in 96-well plates with 10 *μ*M of NB7M or vehicle and cell viability of the TCA fixed treated and untreated cells assessed after 48 h with sulphorhodamine-B (SRB) solution and absorbance read at 515 nM. (**B**) Dose-dependent effect of NB7M on ovarian cancer cells in NCI_60_ screen (see (**A**) and Supplementary Information).

NB7M proved to be highly and dose-dependently cytotoxic for all five cancer cell lines studied, including ovarian cancer cell lines SKOV-3 and OVCAR-3 (60–70% cytotoxicity at 2.5 *μ*M) (Figure 1B). In contrast, TCL-1 and HTR-8 (trophoblasts) were not affected at 5 *μ*M NB7M. As these control cell lines possess a similar metabolic rate as cancer cells in this screen, NB7M apparently showed selective cytotoxicity against cancer-derived cells. To further evaluate the tumour-type selectivity of NB7M, the effect of this compound at 10 *μ*M was screened in an NCI_60_ cell line growth/viability assay (http://dtp.nci.nih.gov/screening.html, Figure 2A). NB7M at 10 *μ*M proved to significantly reduce the viability of colon cancer cells (COLO205, HCT-116, HCT-15, KM12 and SW620), breast cancer (NCI/ADR-RES, MCF7), ovarian cancer (IGROV1, OVCAR-3, OVCAR-8), leukaemia (CCRF-CEM, HL-60, K-562, MOLT-4), renal cancer (ACHN, CAKI-1, SN12C, TK-10), melanoma (LOX IMVI, M14, MALME-3M, SK-MEL-2, SK-MEL-28), prostate (DU145) and non-small lung cancer cells (NCI-H23) cells. In contrast, several non-small lung cancer cell subtypes (EKVX, NCI-H322M and NCI-H226) and a breast cancer cell line (BT-549) were resistant to NB7M treatment (Figure 2A). In the same assay the dose-dependent effect of various concentrations of NB7M (10 nM–100 *μ*M) on a panel of ovarian cancer cell lines was compared (Figure 2B). The growth of IGROV1, OVCAR-3, OVCAR-5 was highly affected by 10 *μ*M of the drug (as shown for OVCAR-3 in the cytotoxicity assay performed in our laboratory) and OVCAR-8 and OVCAR-4 responded strongly to treatment, whereas SKOV-3 revealed less but still significant reduction in growth compared to the other five lines tested by the National Cancer Institute (NCI). Taken together, the NCI screen (http://dtp.nci.nih.gov/screening.html) and our cytotoxicity assays suggest NB7M to be highly and specifically detrimental to cell lines derived from certain tumour types, including ovarian cancer, but less effective against other tumour types and marginally cytotoxic for control cell lines (e.g., TCL-1, HTR-8; Figure 1B and MRC-5 fibroblasts, Singh *et al*, 2008).

### NB7M affects the mitochondrial membrane potential of SKOV-3 and causes morphological hallmarks of apoptosis

**Figure 3:**
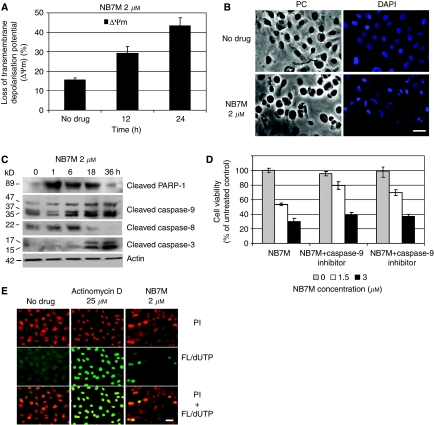
NB7M causes apoptosis in SKOV-3 platinum-resistant ovarian cancer cells. (**A**) Membrane depolarisation analysis after NB7M treatment. SKOV-3 cells were treated for 12 or 24 h with 2 *μ*M NB7M, fixed and stained with DiOC6(3) as described (Materials and Methods). Fluorescence of the single-cell population was measured by flow cytometry and the transmembrane depolarisation potential of the single-cell populations plotted. Ten thousand cells were analysed in each sample. (**B**) Morphological changes following NB7M treatment. SKOV-3 cells were treated for 24 h with 2 *μ*M NB7M, fixed and stained with 4′-6-diamidino-2-phenylindole (DAPI) as described (Materials and Methods) before mounting. Microscopy was carried out (Nikon Eclipse TE2000-E inverted microscope, × 20 objective), and representative images were taken. Bar=10 *μ*M. (**C**) Caspase activation following NB7M treatment. SKOV-3 cells were treated with 2 *μ*M of NB7M for 1, 6, 18 or 36 h. Analysis of the expression of proteins in the lysates of treated and untreated cells was carried out by PAGE and western blot analysis as described (Material and Methods). Primary antibodies against activated caspases-3, -8, -9, and inactivated/cleaved PARP-1 were used. As an internal standard for equal loading, the blots were probed with an anti-*β*-actin antibody. (**D**) Effect of caspase inhibitors on SKOV-3 viability following NB7M treatment. SKOV-3 cells were pre-incubated with specific inhibitor (40 *μ*M) against caspase-3 for 2 h and treated with NB7M (0, 1 or 2 *μ*M) in the continued presence of the inhibitors (40 *μ*M) for an additional 48 h. The MTS viability assay was carried out as described (Materials and Methods). Experiments were performed in triplicates; data are expressed as the mean of the triplicate determinations (X±s.d.) of a representative experiment in % cell viability of samples with untreated cells. (**E**) TUNEL assay. SKOV-3 cells were treated with either 2 *μ*M of NB7M or 25 *μ*M of actinomycin D for 48 h. Labelling of DNA nicks with fluorescein-12-dUTP and chromatin counterstaining with propidium iodide was carried out as described (Materials and Methods). Representative images were taken, apoptotic stain (FL-dUTP, green) and nuclear stain (Pi, red) overlaid; TUNEL-positive nuclei because of DNA fragmentation appear as yellow areas. Bar=10 *μ*M.

To understand the mechanism involved in the cellular response to NB7M treatment, we examined the mitochondrial transmembrane depolarisation potential (ΔYm) of SKOV-3 cells by flow cytometry. NB7M at a concentration of 2 *μ*M caused a rapid loss of ΔYm (29% loss within 12 h; 44% within 24 h) in SKOV-3 cells (Figure 3A). NB7M showed similar reductions in the ΔYm of SMS-KCNR, a chemotherapy-resistant neuroblastoma cancer cell line (Brard *et al*, 2008). Loss of ΔYm due to chemical agents has been reported to be an indicator of onset of early apoptotic events (Petit *et al*, 1995).

In addition, treatment of SKOV-3 cells with NB7M resulted in drastic morphologic changes. Untreated cells or cells treated for 24 h with 2 *μ*M NB7M after fixation and chromatin staining with DAPI were analysed by light and fluorescence microscopy. The population of untreated cells showed a homogenous morphology with nuclei lightly and evenly stained by DAPI (Figure 3B, no drug). In contrast, after treatment with NB7M (2 *μ*M) only ∼20% of cells appeared unaffected, whereas the majority of cells (Figure 3B) showed densely stained nuclear granular bodies of highly condensed chromatin (‘apoptotic bodies’), a classic hallmark of apoptosis (Earnshaw, 1995).

### Induction of apoptosis in SKOV-3 ovarian cancer cells by NB7M

We analysed the activation of cellular markers that are characteristic of apoptosis by immunoblotting. Apoptosis is executed by caspases; initiator caspases (such as caspases-2, -8, -9 and -10) once activated, cleave and activate downstream effector caspases (such as caspases-3, -6 and -7), which are responsible for the cleavage of many intracellular proteins, leading to the morphological and biochemical changes associated with apoptosis (Thornberry and Lazebnik, 1998; Salvesen and Abrams, 2004). NB7M treatment of SKOV-3 cells resulted in the activation/cleavage of initiator caspases-8 and -9 (within 1–6 h) followed by enhanced upregulation of executioner caspase-3 within 18 h (reaching maximal activation at 36 h) (Figure 3C). The activation of proteolytic caspases following drug exposure resulted in the cleavage of PARP-1 (Figure 3C). PARP, a 116 kDa nuclear poly (ADP-ribose) polymerase, is involved in DNA repair (Satoh and Lindahl, 1992), and cleavage of PARP facilitates cellular disassembly and serves as a marker of cells undergoing apoptosis (Oliver *et al*, 1998). The proof that reduction of SKOV-3 viability by NB7M is a direct consequence of the induction of apoptosis is shown in Figure 3D. We used caspase-3 or -9 inhibitors, which were added to the viability assay 2 h before and during the treatment with NB7M. Cytotoxicity of NB7M (at 1 *μ*M) was reduced by ∼60% following addition of the caspase-3 inhibitor and by ∼35% following inhibition of caspase-9.

A TUNEL assay, a common method for detecting DNA fragmentation resulting from apoptotic signalling cascades, was carried out. The assay relies on the presence of nicks in the DNA of apoptotic (and necrotic) cells, which can be identified by a terminal transferase that catalyses the addition of labelled dUTP. SKOV-3 cells were treated with either 2 *μ*M of NB7M or 25 *μ*M of actinomycin D (positive control for drug-induced DNA fragmentation) for 48 h. Nuclei were counterstained with propidium iodide. TUNEL-positive nuclei were identified by yellow spots, resulting from an overlay of the image with apoptotic staining (FL-dUTP, green) and nuclear staining (Pi, red). As shown in Figure 3E, a significant number of cells after NB7M treatment were TUNEL-positive indicating fragmented DNA.

### NB7M treatment causes MAPK activation and suppression of prosurvival markers in SKOV-3 ovarian cancer cells

**Figure 4:**
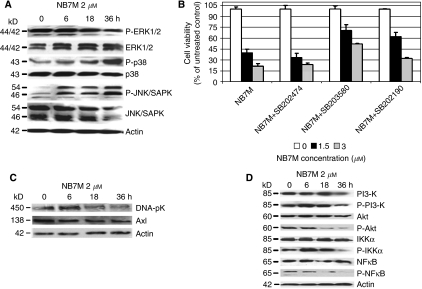
Expression of prosurvival markers and MAPKs in SKOV-3 following NB7M treatment; effect of MAPK inactivation on cell viability. (**A**) Activation of MAPKs. SKOV-3 cells were treated with 2 *μ*M of NB7M for 6, 18 or 36 h. Analysis of the expression of proteins in the lysates of treated and untreated cells by PAGE and western blot analysis was carried out as described (Material and Methods), using primary antibodies against pro- and activated/phosphorylated (P-) SAP/JNK, p38 and ERK1/2. As an internal standard for equal loading, the blots were probed with an anti-*β*-actin antibody. (**B**) Effect of p38 MAPK inactivation on cell viability. SKOV-3 cells were pre-incubated with specific inhibitors (40 *μ*M) against p38 MAPK for 2 h and treated with NB7M (0, 1 or 2 *μ*M) in the continued presence of the inhibitors for an additional 48 h. The MTS viability assay was carried out as described (Materials and Methods). Experiments were performed in triplicates; data are expressed as the mean of the triplicate determinations (X±s.d.) of a representative experiment in % cell viability of samples with untreated cells. (**C**) Effect of NB7M on DNA-pK and Axl. SKOV-3 cells were treated with 2 *μ*M of NB7M for 0, 6, 18 or 36 h. Analysis of the expression of proteins in the lysates of treated and untreated cells by PAGE/western blot analysis was carried out using primary antibodies against DNA-pK and Axl proteins. (**D**) Inactivation of survival signalling proteins and transcription factor proteins: SKOV-3 cells were treated with 2 *μ*M of NB7M for 6, 18 or 36 h. Analyses of the expression of proteins in the lysates of treated and untreated cells by PAGE/western blotting were carried out using primary antibodies against pro- and activated/phosphorylated PI-3K, STAT-3, IKK*α* or NF-*κ*B.

To define key signalling responses of SKOV-3 cells to treatment with NB7M, we analysed the expression and activation/phosphorylation of cellular markers involved in prosurvival or pro-apoptotic signalling. Immunoblotting of PAGE-separated cellular lysates revealed that NB7M (at 2 *μ*M) caused a rapid, strong, and sustained activation of p38 and JNK MAPK (Figure 4A). Both MAPKs are crucial factors in signalling cascades responding to inflammatory cytokines, stress, UV light, osmotic shock, cytotoxic drugs and diverse pro-apoptotic stimuli (Pearson *et al*, 2001). Upregulation of activated p38 and JNK MAPKs resulted in slight downregulation of the basal level of inactive JNK and p38 (Figure 4A). In addition, expression of the phosphorylated/activated form of ERK1/2 in SKOV-3 was downregulated upon NB7M treatment (2 *μ*M), whereas inactive ERK1/2 remained at high level in untreated or treated SKOV-3 cells. Both ERK 1 and 2 (p44 and p42 MAPKs) generally participate in a protein kinase cascade that plays a critical role in the regulation of cell growth and differentiation and can be found activated in their role as survival factors as well as in apoptotic events (Pearson *et al*, 2001; Ahmed-Choudhury *et al*, 2006).

We directly addressed the role of p38 activation in the lethal response of SKOV-3 cells to NB7M by performing the viability assay in the presence of MAPK inhibitors (p38/SB203580, p38/SB202190 and negative control, SB202474, Figure 4B). Inhibitors (40 *μ*M) were added to the viability assay 2 h before and during the treatment with NB7M (1.5 or 3 *μ*M). A significant suppression of NB7M cytotoxicity was achieved by interfering with p38 MAPK signalling (∼40 to 60% restoration of viability at 1.5 *μ*M NB7M depending on the inhibitor used) (Figure 4B).

In addition to the activation of pro-apoptotic kinases, NB7M excerted inhibitory effects on various prosurvival kinases. Immunoblotting experiments indicated that downregulation of DNA-Pk and Axl expression in SKOV-3 cells upon NB7M treatment occurred within 18 h of treatment (Figure 4C) and revealed a time-dependent downregulation of the activation/phosphorylation of various prosurvival proteins such as PI-3k (phosphatidylinositol 3-kinase), Akt, IKK*α* and transcription factor NF-*κ*B (Figure 4D).

### NB7M effect on cell proliferation and cell-cycle progression

**Figure 5:**
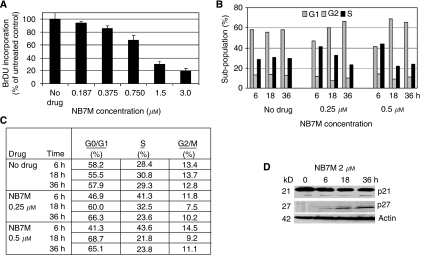
Effect of NB7M on proliferation and cell-cycle progression of SKOV-3 cells. (**A**) Cell Proliferation/BrdU incorporation. SKOV-3 cells were treated with NB7M (0, 0.187, 0.375, 0.75, 1.5, 3 *μ*M) for 48 h. The proliferation assay was carried out as described (Materials and Methods). Experiments were performed in triplicates; data are expressed as the mean of the triplicate determinations (X±s.d.) in % cell proliferation of untreated cells. (**B** and **C**) Cell-cycle analysis by FACS. SKOV-3 cells were treated with various NB7M (250 or 500 nM) for 6, 18 or 36 h. Cell-cycle analysis of treated and untreated cells was carried out as described (Materials and Methods). Data are presented as the relative fluorescence intensity of cell subpopulations in a bar chart (**B**) or table (**C**). (**D**) Expression of cyclin-dependent kinase inhibitors in NB7M-treated SKOV-3 cells. Expression of p21 and p27 inhibitors in NB7M and vehicle-treated SKOV-3 cells were analysed by western blotting of lysates and probed with the appropriate primary and secondary antibodies.

As described in the previous sections, NB7M is a cytotoxic agent that activates apoptotic processes in SKOV-3 ovarian cancer cells. To investigate if NB7M affects the proliferation of SKOV-3 cells (particularly at drug concentrations when viability is not affected or partially reduced), we performed a BrdU incorporation assay. In this assay, BrdU incorporation into replicating DNA is detected by an antibody–peroxidase conjugate allowing a colorimetric reaction with the colour intensity directly representing cell proliferation. NB7M dose-dependently reduced SKOV-3 proliferation (Figure 5A). At a drug concentration of 1.5 *μ*M (for 48 h) proliferation of treated SKOV-3 was inhibited by 70% as compared to untreated cells. Even at drug concentrations in the nano-molar range BrdU incorporation into the DNA was reduced. Thus, NB7M is a potent pro-apoptotic agent as well as an antiproliferative agent.

Cell-cycle analysis of SKOV-3 cells after NB7M treatment at subcytotoxic concentrations (250 and 500 nM) revealed a significant increase in the G1-phase cell population and reduction in S-phase cells compared to untreated control, while the G2 cell population remained largely unaffected within 18–36 h of treatment (Figure 5B and C). Interestingly, 6 h of treatment temporarily led to a decrease of cells in the G1-phase and an increase in S-phase. This indicated that proliferation and S-phase progression was adversely affected within 6 h of treatment, while a block of cell-cycle progression in G1 in this non-synchronised culture unfolded within 18 h of treatment. Immunoblotting revealed that NB7M treatment of SKOV-3 affected the expression of two prominent members of the Cip/Kip family of cyclin-dependent kinase (CDK) inhibitors, which are key regulators involved in cell growth *in vivo*, *in vitro*, in tumour and normal tissues (Stillman, 1996; Albrecht *et al*, 1998; Pines, 1999): P27 (kip1) CDK was upregulated and p21 downregulated upon NB7M treatment (Figure 5D). These preliminary observations suggest further studies on the specific antiproliferative mechanisms of NB7M in cancer cell lines (e.g., ovarian tumour).

## Discussion

NB7M is a novel synthetic indole ethyl ITC designed from 7Me-IEITC, a potent cytotoxic agent in its own right (Singh *et al*, 2007, 2008). Unprotected indole nitrogen groups of 7Me-IEITC can potentially cause chemical degradation reactions. Accordingly, NB7M was synthesised by ^t^Boc-protection of the indole nitrogen of 7Me-IEITC; the addition of an acid-labile protecting group (tert-butyl carbamate) on the secondary nitrogen is most likely to enhance the stability of the indole ethyl ITC. We recently reported that NB7M shows potent apoptotic and inhibitory effects on the cell cycle in nervous system cancer cell lines (Brard *et al*, 2008).

In this study, the cytotoxicity of NB7M in well-characterised human solid tumour cancer cell lines (ovarian, prostate, skin and pancreas) and two human control cell lines with similar growth rate (TCL-1/immortalised trophoblasts and HTR-8-SVneo/first-trimester cytotrophoblasts with extended lifespan) was defined. NB7M showed no adverse effect on TCl-1 and HTR-8 at a concentration (5 *μ*M) that was highly cytotoxic to the five cancer cell lines. Given the differential responses of controls versus cancer cells tested, the effect of NB7M on cell growth was screened by the NCI Developmental Therapeutics Program (DTP) against a panel of 60 cancer cell lines, which broadly represent human tumours of colon, ovarian, lung, melanoma, leukaemia, renal, prostate and central nervous system. NB7M showed potent activity against most tumour types. Notable exceptions were non-small cell lung cancer cell subtypes NCI-H322H, HOP-62, EKVX and A549. The compounds minimal activity against several non-small lung cancer cells is reminiscent of data retrieved after the treatment by naturally occurring ITCs (PEITC and BITC) causing cell death of A549 non-small lung cancer cells only at higher doses (Fang and Hwa, 2004). The cytotoxicity toward SKOV-3 ovarian cancer cells, however, is several fold higher when NB7M is used instead of BITC and PEITC (this study, Kalkunte *et al*, 2006; Satyan *et al*, 2006). The DTP at the NCI maintains a public accessible database of cytotoxic drugs; a comparison analysis (COMPARE) of known cytotoxic drugs versus NB7M indicated that NB7M is more potent than a variety of clinically relevant drugs, such as cisplatin, oxaloplatin, seliciclib, CNDAC, 5-FU and cyclophosphamide, in most of the NCI_60_ cell lines but is less potent than docetaxel, adriamycin and gemcitabine.

Our *in vitro* investigations have shown that NB7M and its synthetic parent compound 7Me-IEITC show overlapping cytotoxic effects with ovarian cancer cell lines OVCAR-3 and SKOV-3 being more sensitive to NB7M as compared to 7Me-IEITC (present manuscript; Singh *et al*, 2008). This also applies to several neuroblastoma cell lines, while the viability of lung fibroblasts or trophoblast cell lines by either compound is only marginally affected (Singh *et al*, 2007; Brard *et al*, 2008). These overlapping effects are not surprising because 7Me-IEITC, due to conversion by hydrolytic enzymes such as amidases, may be a metabolite of the N4-protected NB7M, similar to conversion of N4-protected sapacitabine into gemcitabine and ara-C in cancer cells (Serova *et al*, 2007). Nevertheless, differences in the degree of cytotoxic action by these related compounds could be explained by the unique stereo-electronic properties of NB7M due to a carbamate group not present in 7Me-IEITC, which may offer additional interactions with target proteins. In addition, the introduction of a tertiary butyl carbamoyl substituent of the indole nucleus of 7Me-IEITC most likely endowed NB7M with resistance to intramolecular or intermolecular reactions (e.g., between the indole nitrogen with the ITC group). Finally, the fact that cells may accumulate and retain a higher concentration of NB7M (because it is more lipophilic than 7Me-IEITC) might explain the differences in drug cytotoxicity.

NB7M and its precursor 7Me-IEITC showed a striking similarity in the expression and/or activation of a multitude of molecular targets, including DNA-PK, Axl, PI-3K, Akt; the MAPKs ERK1/2, p38, JNK; NF-*κ*B and the CDK inhibitor p27 (present manuscript; Singh *et al*, 2007; Brard *et al*, 2008; Singh *et al*, 2008). Many types of tumours are associated with activated oncogenic kinases. These kinases play two complementary roles; they stimulate signalling pathways that enable cells to function independent of their environment and they cause tumour cells to become resistant to genotoxic therapies (Hanahan and Weinberg, 2000). For example, Axl is known to bind the vitamin K-dependent protein growth-arrest-specific gene 6 (Gas6) and to activate PI-3K and its downstream targets S6K, Akt and NF-*κ*B (Crosier and Crosier, 1997; Demarchi *et al*, 2001). The serine/threonine kinase, Akt, and its family members are amplified or their activity is constitutively elevated in human carcinomas such as breast, pancreatic, ovarian, brain, prostate and gastric tumours (Nicholson and Anderson, 2002). Drugs such as NB7M that can block Akt, PI-3K, survival kinase IKK*α* and transcription factor NF-*κ*B activities, which are known to contribute to tumour growth by promoting cell-cycle entry, cell proliferation, cell migration, or antiapoptotic responses, and are implicated in resistance to radio- and chemotherapy (Kip *et al*, 2002; Greten and Karin, 2004), are potential cancer therapeutics.

As it is a direct downstream target of PI-3K, Akt is a key oncogenic survival factor and can inactivate a broad panel of critical pro-apoptotic molecules such as p38 MAPK, Bad, caspase-9, the Forkhead transcription factor FKHRL1, GSK3-*β*, cell-cycle inhibitors p21 and p27, and tumour suppressors p53 and TSC2 (Zhou *et al*, 2001; Inoki *et al*, 2002; Blain and Massague, 2002; Liao and Hung, 2003). Activation of Akt has been shown to induce resistance to apoptosis induced by a number of drugs (Page *et al*, 2000). Activation of the PI-3K/Akt pathway has been linked to cisplatin resistance in ovarian cancer cell lines (Lee *et al*, 2005). Consequently, our observations that NB7M inactivates the PI-3K pathway and downstream factors IKK*α* and NF-*κ*B suggests that NB7M could be used to enhance the effectiveness of chemotherapy of drug resistant tumours. Several natural compounds and synthetic drugs that are able to inhibit the IKK/NF-*κ*B activation pathway have been shown to either prevent cancer or to inhibit cell growth in animal models (Bharti and Aggarwal, 2002), and we suggest further exploration of potential chemotherapeutic properties of NB7M in an ovarian cancer animal model.

This report also shows that NB7M, in addition to its selective cytotoxic properties, at subcytotoxic concentrations acts as a potent inhibitor of SKOV-3 ovarian cancer cell proliferation. NB7M treatment affects cell-cycle checkpoints in G1-phase, causing reduction in the progression of cells into S- and G2/M-phase. Blocking the progression of dividing cells to G2/M-phase also reduces the possibility of DNA repair and thereby leads to increased cell death, counteracting/bypassing resistance mechanisms. Similarly, another plant-derived molecule, Guggulsterone, caused cell-cycle arrest in S-phase by the suppression of cyclin D1 and cdc2 and increased CDK inhibitor p21 and p27 expression in a wide variety of human tumour cell types (Shishodia *et al*, 2007). Apparently, transformed cells can be more sensitive to CDK inhibition because of the fact that components of the cell-cycle machinery are frequently altered in human cancer (Hartwell and Kastan, 1994; Gladden and Diehl, 2003), and thus, can be specifically targeted. Even though not the objective of this report, future studies will examine the effects of NB7M on specific cell-cycle checkpoints in synchronised ovarian cancer cells. Targeting cell-cycle checkpoints has been suggested as an alternative approach to anticancer therapies (Shapiro and Harper, 1999; Mazumder *et al*, 2004), and NB7M at subcytotoxic concentrations has the potential to selectively interfere with the progression of cancer cell proliferation.

In summary, this report suggests that NB7M is a potent growth-suppressing agent to cell lines derived from ovarian cancers and a potential therapeutic drug to treat such tumours *in vivo*.

## Supplementary Material

Supplementary On-Line Materials
