# Supplementary material for: Isothiocyanate NB7M causes selective cytotoxicity, pro-apoptotic signalling and cell-cycle regression in ovarian cancer cells
Source: Br J Cancer. 2008 Nov 11;99(11):1823–31. doi: 10.1038/sj.bjc.6604778 (PMC2600706; doi:10.1038/sj.bjc.6604778)
Supplement: Supplementary On-Line Materials [file 6604778x1.doc]

**Supplementary On-Line Materials.**

**Synthesis and structural characterization of NB7M**

Chemicals and reagents were purchased from Aldrich (Milwaukee, WI). **NB7M** was synthesized as per the scheme described previously. 7-Methyl-indole ethyl isothiocyanate (7Me-IEITC) (Singh *et al,* 2007) (1eq.) was treated with di-tertiary butyl carbamate (3.0 eq.) in dichloromethane and triethyl amine (6 eq.) in the presence of a catalytic amount of dimethylamino pyridine (DMAP) in anhydrous dichloromethane (25mL) at 0^o^C for 96 h. The reaction mixture was diluted with dichloromethane and washed with 5% NaHCO_3_ aqueous solution and finally with H_2_O. The organic layer was collected, concentrated under reduced pressure and purified by preparative thin layer chromatography by eluting with EtOAC:Hexane (20:80). The purified compound, **NB7M** a viscous oil, was characterized by Mass spectrometry (API-150, positive ion electrospray ionization source) (**Figure A1**). Purity and stability was ascertained by high performance liquid chromatography (HPLC) under two different solvent conditions (**Figure A2**).

**HPLC conditions**:

Analytical quantification of **NB7M** was achieved using high performance liquid chromatography (HPLC; Waters, Milford, MA, USA) using a C18 Luna column (4.6 x 150 mm; 5 µm; Phenomenex, Torrance, CA, USA). **NB7M** (40 µL) was injected into the column and a gradient elution was used for fractional separation with two solvents at a flow rate of 1.5 ml/minute. Solvent A consisted of 10% MeOH in H2O adjusted to pH 3.5 with formic acid. Solvent B consisted of 20% H_2_O (pH 3.5), 20% MeOH, and 60% acetonitrile. The gradient consisted of 0 min-90% A, followed by 15 min-0% A, 20 min-0% A, 30 min-100 % A, and held at 100% A for a final 35 min. Five minutes of equilibration at 100% A was performed before and after each injection. **NB7M** identified by scanning by photo diode array (PDA) detector. **NB7M** used in this study was found to have a retention time (RT) of 18.16 minutes and a purity of 98%.

**Stability of NB7M in solution**:

Stability of **NB7M** was assessed by comparing two samples at concentration of 5 and 50 µg/mL in DMSO. Analyte samples were stored at 10 °C for 72 h and -20 °C for 12 weeks. Before each analysis samples were freeze-thawed three times and then analyzed. The stability of **NB7M** in DMSO was investigated by comparing the peak area under curve against freshly prepared standard stock solutions. Using these methods, the stored **NB7M** samples exhibited no significant changes in the purity compared to freshly prepared **NB7M** solutions.

***Description of NCI _60_ cell line in vitro Screening and Methodology***

**NB7M** was screened in the DTP-NCI_60_ cell line panel at a single dose (10μM) under the *in vitro* Cell Line screening Project (IVCLSP) and growth percent of treated cells estimated relative to control (100%) [DTP/NCI web site (http://dtp.nci.nih.gov)]. The screening is a two-stage process beginning with the evaluation of all compounds against the 60 cell lines at a single dose of 10 µM. The [output](http://dtp.nci.nih.gov/branches/btb/onedose_interp.html) from the single dose screen is reported as a mean graph and is available for analysis by the COMPARE program. Compounds which exhibit significant growth inhibition are evaluated against the 60 cell panel at five concentration levels.

Typically, the human tumor cell lines of the cancer screening panel are grown in RPMI 1640 medium containing 5% fetal bovine serum and 2 mM L-glutamine. For a typical screening experiment, cells are inoculated into 96 well microtiter plates in 100 µL at plating densities ranging from 5,000 to 40,000 cells/well depending on the doubling time of individual cell lines. After cell inoculation, the microtiter plates are incubated at 37 °C, 5 % CO_2_, 95 % air and 100 % relative humidity for 24 h prior to addition of experimental drugs. After 24 h, two plates of each cell line are fixed *in situ* with TCA, to represent a measurement of the cell population for each cell line at the time of drug addition (Tz). Experimental drugs are solubilized in dimethyl sulfoxide at 400-fold the desired final maximum test concentration and stored frozen prior to use. At the time of drug addition, an aliquot of frozen concentrate is thawed and diluted to twice the desired final maximum test concentration with complete medium containing 50µg/ml gentamicin. Additional four, 10-fold or ½ log serial dilutions are made to provide a total of five drug concentrations plus control. Aliquots of 100 µl of these different drug dilutions are added to the appropriate microtiter wells already containing 100 µl of medium, resulting in the required final drug concentrations. Following drug addition, the plates are incubated for an additional 48 h at 37°C, 5 % CO_2_, 95 % air, and 100 % relative humidity. For adherent cells, the assay is terminated by the addition of cold TCA. Cells are fixed *in situ* by the gentle addition of 50 µL of cold 50 % (w/v) TCA (final concentration, 10 % TCA) and incubated for 60 minutes at 4 °C. The supernatant is discarded, and the plates are washed five times with tap water and air dried. Sulforhodamine B (SRB) solution (100 µl) at 0.4 % (w/v) in 1 % acetic acid is added to each well, and plates are incubated for 10 minutes at room temperature. After staining, unbound dye is removed by washing five times with 1 % acetic acid and the plates are air dried. Bound stain is subsequently solubilized with 10 mM trizma base, and the absorbance is read on an automated plate reader at a wavelength of 515 nm. For suspension cells, the methodology is the same except that the assay is terminated by fixing settled cells at the bottom of the wells by gently adding 50 µl of 80 % TCA (final concentration, 16 % TCA). Using the seven absorbance measurements [time zero, (Tz), control growth, (C), and test growth in the presence of drug at the five concentration levels (Ti)], the percentage growth is calculated at each of the drug concentrations levels. Percentage growth inhibition is calculated as:

[(Ti-Tz)/(C-Tz)] x 100 for concentrations for which Ti>/=Tz

[(Ti-Tz)/Tz] x 100 for concentrations for which Ti<Tz.

Three dose response parameters are calculated for each experimental agent. Growth inhibition of 50 % (GI50) is calculated from [(Ti-Tz)/(C-Tz)] x 100 = 50, which is the drug concentration resulting in a 50% reduction in the net protein increase (as measured by SRB staining) in control cells during the drug incubation. The drug concentration resulting in total growth inhibition (TGI) is calculated from Ti = Tz. The LC_50_ (concentration of drug resulting in a 50% reduction in the measured protein at the end of the drug treatment as compared to that at the beginning) indicating a net loss of cells following treatment is calculated from [(Ti-Tz)/Tz] x 100 = -50. Values are calculated for each of these three parameters if the level of activity is reached; however, if the effect is not reached or is exceeded, the value for that parameter is expressed as greater or less than the maximum or minimum concentration tested.

**Figure A1**: Electro-Spray (ES) Mass Spectrum of **NB7M** [MH^+^=317] in positive ion mode.


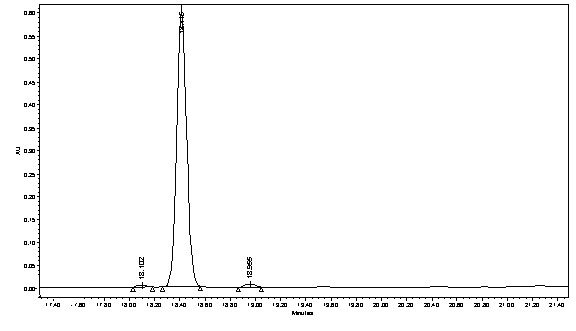


**Figure A2**: Purity analysis of **NB7M** by high performance liquid chromatography (HPLC): 98% at 209nm (retention time: 18.16 min).
